# Does LigaSure^™^ reduce fluid drainage in axillary dissection? A randomized prospective clinical trial

**DOI:** 10.3332/eCMS.2007.61

**Published:** 2007-11-29

**Authors:** M Antonio, T Pietra, LG Domenico, D Massimo, R Ignazio, N Antonio, C Luigi

**Affiliations:** 1 Department of Experimental Oncology and Clinical Application, Faculty of Medicine and Surgery, University of Palermo, Palermo, Italy; 2 Department of Surgical Oncology, Breast Unit, La Maddalena Cancer Centre, Via San Lorenzo no. 312/d CAP, 90146 Palermo, Italy

**Keywords:** Breast carcinoma, breast cancer surgery, axillary dissection, seroma, drain, haemostasis, LigaSure

## Abstract

**Background:**

Axillary lymph node dissection (ALND) is an integral part of breast cancer treatment. It is required in about 40–50% of patients. The placement of a drain in the axilla after an operation is current surgical practice. Short surgical stay programmes increase operating efficiency and reduce medical care costs, without compromising quality of care. LigaSure^™^ is a new haemostatic device that uses bipolar energy to seal vessels. The aim of this study is to determine whether axillary dissection with LigaSure^™^ reduces the time of wound drainage, the duration of surgical intervention and the volume of drainage after treatment.

**Patients and methods:**

This study is a prospective randomized controlled trial. A total of 100 women with breast cancer who needed axillary dissection were randomized into the LigaSure^™^ or conventional axillary dissection group. Levels I to III lymph node dissection was performed. A closed suction drain was always placed in the axilla and removed after 6–8 days or when fluid amount was <60 cc in the previous 24 hours.

**Results:**

There were no significant differences between the two groups when considering the duration of surgical procedure: average duration was 70.7 ± 24.66 minutes for LigaSure^™^ patients, while in the conventional dissection group the mean was 70.6 ± 22.47 minutes (p=0.98). Total amount of drained fluid was 624.49 cc in the LigaSure^™^ axillary dissection group and 792.96 in the conventional ALND group; this difference did not achieve statistical significance (p=0.09); the duration of draining was also similar, with no statistical difference (p=0.15).

**Conclusions:**

The present study did not show clear advantages in LigaSure^™^ use for ALND, although it represents a good haemostatic device, especially in abdominal surgery.

## Introduction

Axillary lymph node dissection (ALND) is an integral part of breast cancer therapy. It is required for patients with clinically positive lymph nodes and for those with clinically negative lymph nodes but with positive sentinel nodes. ALND is necessary in 40–50% of patients with breast cancer. Postoperative complications include accumulation of serosanguinous fluid producing a seroma with a reported incidence of 5–80%[1–4]. The incidence of seroma has been shown to correlate with breast size, hypertension [5], presence of malignant nodes in the axilla, number of malignant nodes, previous surgical biopsy and use of heparin [6].

The origin of seroma is multi-factorial: it includes lymphorrhea from severed lymphatic vessels, local inflammation, surgically created dead space and use of electrocautery [7–9]. Currently accepted surgical practice for the prevention of seromas consists of insertion of a drain during the operation. A drain requires careful management and is usually removed when fluid output is reduced to approximately 40 cc per day, usually from seven to 14 days after the operation. A technique that would allow earlier drain removal or eliminate drains altogether might decrease morbidity and costs and enhance the patient’s rehabilitation and satisfaction.

LigaSure^™^ (Valleylab, Boulder, CO, USA) is a device of bipolar haemostasis with automatic ‘switch-off’ when impedance reaches a critical level. LigaSure^™^ seals blood vessels using a precise amount of energy and pressure that permanently changes the collagen and elastin pattern within the vessel wall. It ensures complete coagulation with minimal thermal spread and limited tissue charring [10,11]. The aim of this study was to determine whether axillary dissection with LigaSure^™^ reduces the duration and amount of fluid drainage.

## Methodology

This prospective randomized controlled trial was conducted at the Breast Unit of La Maddalena Cancer Centre in Palermo between January 2005 and December 2006. A total of 100 women with breast cancer who required axillary dissection were recruited for the trial. Patients were randomized to the LigaSure^™^ or conventional axillary dissection group at the time of surgery by closed envelope allocation; no stratification was used.

All axillary dissections were done through an incision that followed a natural skin crease in the axilla. All branches of the axillary vein were divided and ligated at the level of the thoracoacromial vessels. The long thoracic and the thoracodorsal neurovascular bundles were preserved at all times. Level I to III lymph node dissection was performed routinely. A closed suction drain was placed in the axillary fossa in all cases. In the LigaSure^™^ group both fat dissection and vessel sealing were performed using the medium-sized forceps. The drain was removed post-operatively from the 6–8th day or when the total drainage was <60 cc during the previous 24 hours. Informed consent was obtained pre-operatively in accordance with the ethical standards of the Helsinki Declaration of 1975. All patients gave their consent after understanding the aim of the study, the different kinds of surgical procedures and the post-operative morbidity.

The primary end point was whether the use of LigaSure^™^ reduced the time needed for wound drainage compared to the control group, and the duration of surgical intervention. The secondary end point was whether LigaSure^™^ reduced the volume of drainage after treatment. Safety was determined by comparing the incidence of adverse events. The person recording the volume of fluid collected from drains, whether a nurse, patient, family member or a member of the research staff, was blind to treatment assignment.

Results are expressed as mean ± standard deviation or number (%). Group differences for continuous variables were assessed by Fisher’s exact test. Group differences for other categorical variables were assessed by the chi-square test. Statistical significance was determined by using the alpha level of 0.05 and the two-sided *t* test.

## Results

One hundred patients were enrolled in the study and all were available for evaluation of the predefined end points and safety assessments. Patients’ characteristics are listed in Table 1. There were no significant differences in T and N stage between the two groups: the p values were 0.36 and 0.13, respectively. The distribution of performed surgical procedures was similar in LigaSure^™^ and control patients: quadrantectomy with ALND dissection was 32 (64%) versus 22 (44%); modified radical mastectomy was 18 (36%) versus 28 (56%) with p=0.44.

Concomitant diseases (hypertension, diabetes mellitus and obesity) and a previous protocol of anti-blastic therapy were also considered and the results showed no significant differences between the two groups.

To examine the hypothesis that the number of lymph nodes removed could influence the amount of lymphorrhea, the average of total nodes removed from each group was recorded and it was found that in the LigaSure^™^ group the mean was higher than in the control group, 17.98 ± 5.52 versus 15.34 ± 5.84 (p=0.02). No significance is reported for number of neoplastic nodes: p=0.38. The results of the trial are shown in Table 2.

We did not find any statistically significant differences between the two groups when we considered the duration of the surgical procedure: average duration was 70.7 ± 24.66 minutes for LigaSure^™^ patients, while in the conventional dissection group, the mean was 70.6 ± 22.47 minutes (p=0.98). Total amount of drained fluid was 624.49 cc in the LigaSure^™^ axillary dissection group and 792.96 cc in the conventional axillary dissection group; this difference did not reach statistical significance (p=0.09); the duration of drain was also similar with no statistical difference (p=0.15).

Seroma occurred in 22 patients operated with LigaSure^™^ versus nine patients in which a traditional axillary dissection was performed. The p value recorded for this parameter was statistically significant (0.005). But when the average of fluid drained by punctures of the axillary region and the number of aspirations were considered, there was no significant difference.

A low rate of surgical complications in both groups was recorded: only one patient had a wound dehiscence when LigaSure^™^ was employed, while four dehiscences and one wound haemorrhage that required another operation were observed in the control group. Neither dehiscence nor postoperative haemorrhage was statistically significant (p=0.17 and 0.32, respectively).

## Discussion

To date, breast cancer treatment can be performed in a day-surgery regimen. Short surgical stay programmes increase operating efficiency and reduce medical care costs, without compromising quality of care [12,13]. Twenty-four-hour surgery stays can reduce average hospital costs by 36% [14]. In any case, there is the disadvantage that in patients who need axillary node dissection, it is necessary to place a suction drain to try to prevent seroma formation.

The rich lymphatic drainage of the breast establishes the tendency for seroma formation within any closed space that results from breast surgery. The closed spaces of quadrantectomy cavities and axillary wounds can harbour seroma. Seroma formation under the skin flaps of axillary wounds impairs the healing process; the skin flaps tend to heal and adhere after 1–3 weeks, as evidenced by diminished drain output [7,15].

It is common practice to remove the drain when fluid output is reduced to approximately 40 cc per day, usually from seven to 14 days after the operation.

Seroma collections developing after drain discharge can be simply managed by percutaneous aspiration. It is usually well tolerated by patients and can be repeated as frequently as necessary to ensure that the skin flaps are densely adherent to the chest wall. Seroma aspiration is necessary in 10–80% of patients who underwent ALND, according to several series [15].

Some authors have compared drainage and no drainage in patients undergoing ALND; closed suction drainage appeared advantageous in decreasing the incidence and degree of seroma [16,17]. Talbot and Magarey considered 90 consecutive patients who had breast cancer undergoing ALND to (1) conventional prolonged closed-suction drainage, (2) two-day short-term drainage and (3) no drainage. In the first group, the drains were removed after a median of nearly ten days, with 73% of cases requiring subsequent seroma aspiration. As expected, the short-term and no-drain groups required more frequent seroma aspirations (86% and 97%, respectively) [18].

It is obvious that home management of drains by patients, especially aged people, can be difficult; therefore, a technique that would allow earlier drain removal or eliminate drains altogether might decrease morbidity and costs and enhance the patient’s rehabilitation and satisfaction. Another important aspect of early discharge is psychological acceptance. At the present time, some trials show that early discharge with drain is well accepted by the patients and does not increase the rate of psychological problems [12,19,20].

Many measures have been attempted to avoid post-operative fluid effusion. Obliteration of dead space by chemical means has been attempted by many authors, with no clear benefit, sometimes followed by adverse effects. For example, sclerotherapy using tetracycline has led to severe pain on introduction of the drug, without any demonstrable long-term benefit [21,22]. Bovine thrombin has also proved unsuccessful in this regard. The employment of fibrin glue was encouraged after studies on animals [23,24], especially rats that underwent mastectomy, but in human studies no significant advantage was seen [25–27]. In a recent work, Johnson and Coll showed a lower rate of seroma formation using fibrin glue alone (36.8%) versus conventional drain placement (45%), but this difference was not significant. Moreover, aspirate volumes were significantly higher in the fibrin glue group (439 versus 121 ml; p=0.0015). These authors conclude that the higher cost involved, cumbersome technique and higher aspirate volumes tend to indicate that there is no advantage in using fibrin glue [28].

Pressure dressings have been attempted to reduce fluid effusion in the axilla after the operation. However, to immobilize the shoulder with a sling or to wrap a loose fitting bandage around the upper arm has shown no significant advantage in terms of seroma formation [3,29]. Moreover this approach carries the risk of possible long-term range of motion limitations and may even increase the risk of lymphedema. The Memorial Sloan Kettering Cancer Centre conducted a clinical trial that randomly assigned 135 patients undergoing ALND to receive a compression dressing for four days or standard wound coverage (all of them had conventional drainage as well). The study found no benefit from compression dressings. Both arms had similar total drainage volumes and drainage catheter duration; the compression arm furthermore had increased need for aspiration (mean number of punctures 2.9 in the compression, versus 1.8 in the standard dressing arm; p<0.1) [30].

Only one study has demonstrated the effectiveness of octreotide administration in the post-operative period after ALND, on amount and duration of lymphorrhea, but more randomized trials are needed to confirm the real advantage of its use [31]. In any case, octreotide administration did not influence the hospital stay, or the duration of drain.

Some authors tested the effectiveness of different surgical cutting devices, such as Ultracision. A pilot study was set up to determine if it could reduce complications of seroma formation.

There was a little difference between the two groups in terms of hospital stay, volume or duration of post-operative drainage or subsequent aspiration of seroma [32].

In order to find a way to reduce complications after axillary dissection, we decided to employ LigaSure^™^. This system offers an excellent method for achieving bloodless dissection of vascular tissues. The combination of localized coagulation with minimal collateral thermal spread makes it an ideal device for surgical procedures. Technically, it is simple, easy to learn and quick to apply [10,11].

Our study was based on the hypothesis that accurate blood and lymphatic vessel sealing was related to minor fluid effusion in the post-operative period. Since LigaSure^™^ seals vascular and lymphatic structures determining permanent changes in collagen and elastin patterns within the wall, we thought that this new haemostatic device could improve post-operative outcomes after ALND in terms of complications.

A retrospective study concerning 187 patients who underwent ALND employing surgical clips or LigaSure^™^ followed by drainage is reported in the literature. This study concluded that the duration of drainage was significantly shorter with LigaSure^™^ but its benefits in term of fluid loss remains to be shown. Furthermore, its employment did not reduce the cost of hospitalization [33].

Also, our prospective study failed its aim: we did not find any clear advantage from its use in axillary node dissection. None of the considered parameters showed any significance in terms of p value. On the contrary, a larger number of seroma formations, after drain removal, were observed in the LigaSure^™^ group than in the control group (22 versus 9; p<0.005). Moreover, the use of LigaSure^™^ is more expensive than the traditional procedure: it requires an initial purchase of a generator at a cost of 21,000 euros and adds an additional cost to the operation (about 223 euros per disposable diathermy forceps). Hence, although it represents a good haemostatic device in abdominal surgery, where it decreases the duration of the operation, we think that its employment is not cost-effective in breast cancer treatment.

## Figures and Tables

**Table 1:**
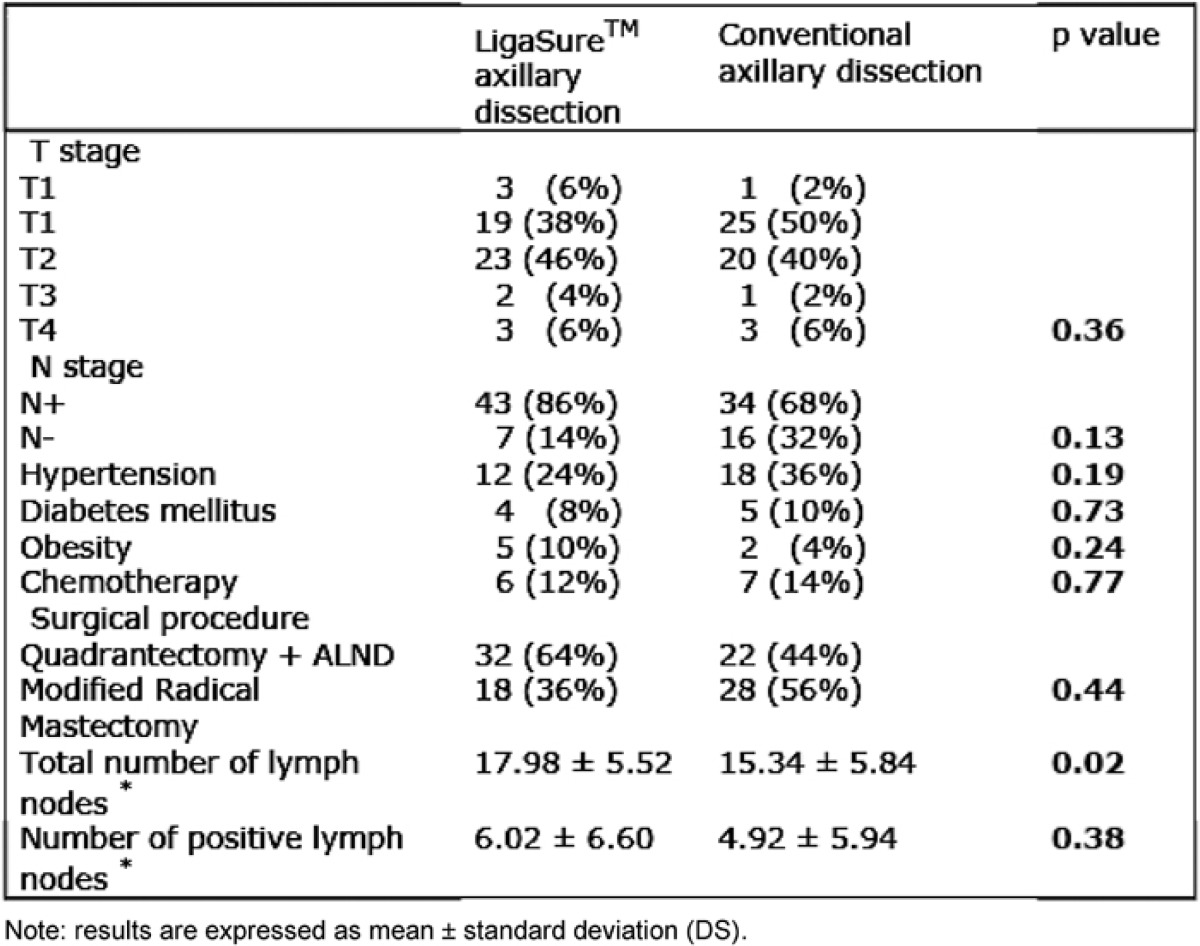
Characteristics of patients randomized to the LigaSure ^™^ group or conventional axillary dissection group

**Table 2:**
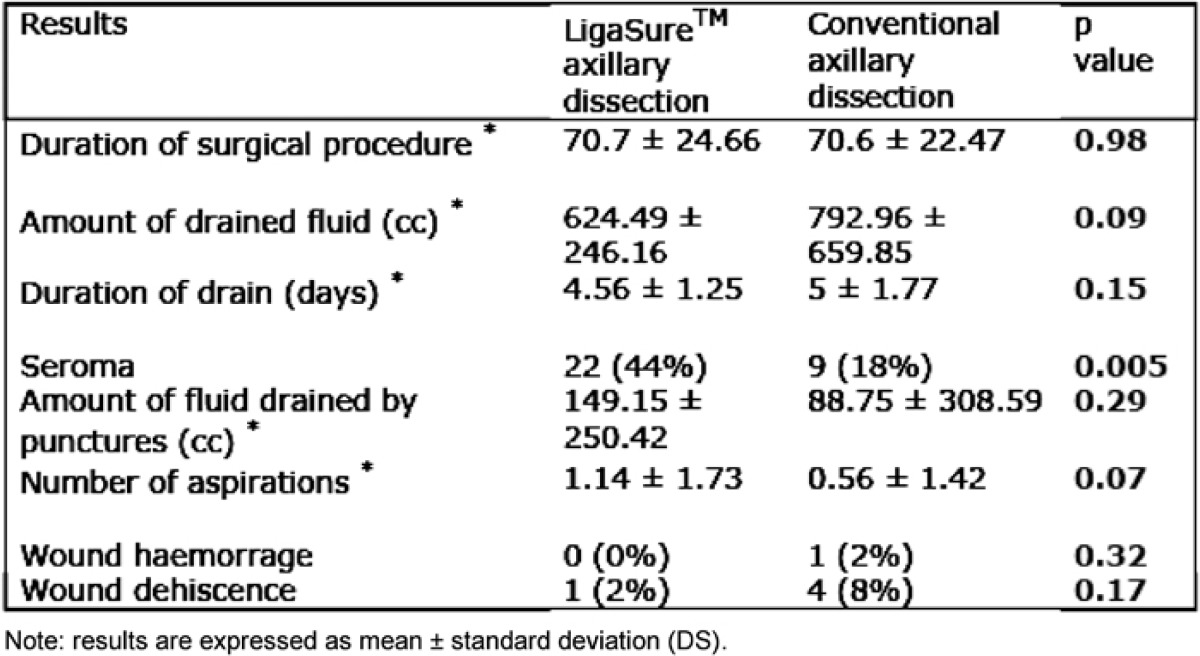
Clinical results of patients who underwent LigaSure^™^ or conventional axillary dissection
